# Identifying TMEM127-deficient pheochromocytomas/paragangliomas via RET overexpression by immunohistochemistry

**DOI:** 10.21203/rs.3.rs-8553860/v1

**Published:** 2026-01-20

**Authors:** Cynthia Estrada-Zuniga, Rui Liang, Bethany Landry, Andrea Alvarez, Hector Gonzalez-Cantu, Viviane Nascimento da Conceicao, Rolando Trevino, David Gius, Sylvia Asa, James Powers, Tamara Prodanov, Anand Vaidya, Rodrigo Toledo, Jean Pierre Bayley, Debbie Cohen, Arthur Tischler, Karel Pacak, Faqian Li, Patricia Dahia

**Affiliations:** The University of Texas Health Science Center at San Antonio; The University of Texas Health Science Center at San Antonio; The University of Texas Health Science Center at San Antonio; The University of Texas Health Science Center at San Antonio; The University of Texas Health Science Center at San Antonio; The University of Texas Health Science Center at San Antonio; The University of Texas Health Science Center at San Antonio; The University of Texas Health Science Center at San Antonio; Case Western Reserve University; Tufts Medical Center; NICHHD; Brigham and Women's Hospital; Vall d'Hebron Hospital Universitari; Leiden University; University of Pennsylvania; Tufts Medical Center; Center for Adrenal Tumors, AKESO; The University of Texas Health Science Center at San Antonio; The University of Texas Health Science Center at San Antonio

**Keywords:** TMEM127, RET, pheochromocytomas, paragangliomas, immunohistochemistry, germline variant, biomarker, VUS

## Abstract

Pheochromocytomas and paragangliomas (PPGLs) are rare, genetically diverse tumors originating from the adrenal medulla or extra-adrenal paraganglia, respectively. Defining a pathogenic variant is critical for patient management and family surveillance, particularly for the 35–40% of patients carrying a germline variant, including those in the *TMEM127* gene. However, determining the functional impact of some variants remains challenging and requires additional testing. We recently reported that loss of *TMEM127* promotes RET accumulation by reducing its degradation. Here, we evaluated RET expression by immunohistochemistry (IHC) as a potential aid to highlight TMEM127 dysfunction in PPGLs. We performed RET IHC in 104 formalin-fixed and paraffin-embedded (FFPE) sections of clinically and genetically diverse PPGLs and applied histochemical scoring (HS) for membrane (MH-S) and cytoplasm (CH-S) staining. Tumors driven by *TMEM127* variants carried the highest RET expression scores (151.8 ± 62), predominantly MH-S, when compared with other PPGL genotypes, including those with *RET* pathogenic disruptions (69.9 ± 96.8, adjusted p = 0.04) or tumors of undefined genotype (40.8 ± 69, adjusted p = 0.0003), reflecting high specificity (100%) and sensitivity (91%). RET membrane immunoreactivity also distinguished PPGLs carrying non-disrupting *TMEM127* variants from variants of uncertain significance (VUS) with likely damaging effects. These results point to high RET membrane expression as a biomarker for pathogenic *TMEM127* and suggest that RET IHC may also assist in interpreting the functional impact of PPGLs carrying *TMEM127* VUS.

## Introduction

Paragangliomas (PGLs) are neural crest-derived tumors of paraganglia arising in the adrenal medulla (also known as pheochromocytomas (PCCs), or other paraganglial tissue of sympathetic or parasympathetic origin, and collectively referred to as PPGLs[1]. PPGLs are recognized as highly heritable tumors, with 35–40% of PPGL cases attributable to germline events that directly influence the surveillance of patients and their relatives at risk of tumor development[2,3]. PPGLs are associated with more than 20 susceptibility genes which align with three molecular clusters that reflect their underlying pathogenesis: pseudohypoxia, also known as cluster 1 (C1), which is further subdivided into C1A, involving disruption of tricarboxylic acid cycle genes, and C1B, related to hypoxia-signaling pathway genes, kinase signaling group (C2), and WNT-altered subtype (C3)[4-6]. This genetic diversity, and the impact that an identifiable pathogenic variant can have on patient diagnosis, surveillance and management, has led to the incorporation of multi-gene panels for the genetic diagnosis of PPGLs[7] [8-13]. Multigene testing has significantly enhanced the detection of genetically actionable variants, leading to improved outcomes in PPGLs[14]. However, these tests also increased the likelihood of detection of variants of uncertain significance (VUS), which are not actionable, and demand further investigation into their role in the disease, for which various methods can be applied[15-17].

Notably, immunohistochemistry (IHC) has proven to be a reliable and broadly accessible assay for evaluating the functional implications of VUS in PPGLs[18]. This approach has been particularly useful in assessing succinate dehydrogenase complex (*SDH*) function. A negative SDHB IHC staining can be interpreted as indicative of a deficient mitochondrial complex II, involving disruption of any one of the *SDHB, SDHC, SDHD* or *SDHA* subunit genes, thus providing valuable insights in clinical management[18, 19]. IHC has also been applied, predominantly in research settings, to evaluate other PPGL genes and often employing the gene product as a biomarker, with variable degrees of success[2, 20, 21].

IHC assays are particularly compelling when employed to complement genetic testing of susceptibility genes for which functional tests are not broadly available outside of a research context. *TMEM127* is one such example. More than half of TMEM127 variants reported in ClinVar are VUS (670 of 1167 recorded variants, 58.2%, as assessed on Dec 19, 2025). Therefore, identification of potential biomarkers associated with *TMEM127* dysfunction could enhance variant interpretation and serve as a valuable adjunct for clinical management and cascade testing in families.

*TMEM127* mutant PPGLs are components of the C2/kinase signaling cluster [5, 6, 22]. We recently uncovered interactions between *TMEM127* and *RET*, the prototypical member of this cluster[23]. Germline *RET* mutations cause familial pheochromocytoma (PCC) as part of Multiple Endocrine Neoplasia Type 2A (MEN 2A) and 2B (MEN 2B), as well as via somatic mutations or more rarely, recombinant fusions, which all lead to constitutive activation of its kinase activity and downstream oncogenic signaling[1, 24]. We found that loss of *TMEM127* function leads to accumulation of the RET protein at the cell surface, by relieving RET from NEDD4-mediated ubiquitination and lysosomal degradation[23]. Accordingly, PPGLs with deficient TMEM127 function showed increased levels of activated RET by analysis of tumor protein extracts. Preliminary analysis of *TMEM127* mutated tumors also suggested strong RET immunoreactivity by IHC[23]. The current study expands upon these earlier findings to evaluate the effectiveness of RET IHC expression as an indicator of TMEM127 dysfunction in PPGLs. By assessing tissues comprising a broad spectrum of genotypes, this study provides insights into the general distribution of RET expression in PPGLs.

## Materials and Methods

### Patient cohort

**Figure 1:**
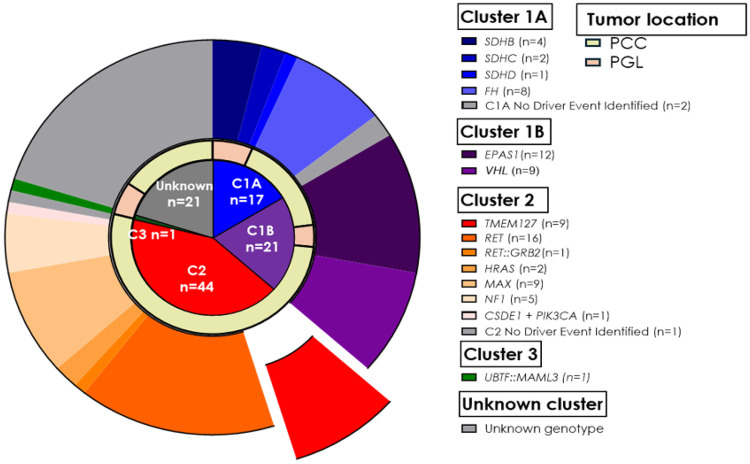
Molecular cluster, genotype and tumor location distribution of the current cohort (C1A: cluster 1A, C1B: cluster 1B; C2: cluster 2; C3: cluster 3; PCC: pheochromocytoma; PGL: paraganglioma

Patients were recruited after providing consent through IRB approval study at the University of Texas Health Science Center at San Antonio and other collaborating institutions, including four A5 Adrenal Alliance affiliated institutions. Each collaborating institution recruited patients through their own IRB approved protocols and provided anonymized samples. Overall, we obtained tissue sections from 89 patients, including 87 individuals diagnosed with PPGL belonging to 81 separate families (Table 1). Although all PPGL genotypes were eligible for recruitment (see details below), we deliberately favored enrollment of samples carrying TMEM127 variants, our main focus, and other samples related to cluster 2 (kinase signaling), especially those from patients carrying pathogenic *RET* variants- including from multiple MEN 2A, MEN 2B and a case with a recombinant fusion (Fig. 1, Table 2). This recruitment bias was intended to favor samples with reportedly higher *RET* transcription to maximize the robustness of the assay[4-6]. Thus, the cohort does not reflect the typical genotype frequency observed in general PPGL series. In addition, tissue sections were available from other cancers (renal cell carcinoma-RCC, gastrointestinal stromal tumor-GIST) detected in two of the patients, and from three relatives of probands who developed other tumors related to PPGL susceptibility genes (Table 1). All histological diagnoses were confirmed by evaluation of hematoxylin and eosin-stained slides.

### Genotyping

DNA sequencing for genotyping was performed on germline and/or tumor samples using exome sequencing, targeted next-generation sequencing panel, or Sanger sequencing, as we previously reported[25, 26]. Genetic driver assignment was based on variant classification as pathogenic/likely pathogenic as previously defined[27, 28] or reported in PPGL literature. VUS and benign/likely benign variants were determined based on the ClinVar database (accessed December 4, 2025).

### Loss of Heterozygosity (LOH) analysis

LOH was determined by assessing the variant allele imbalance in tumor DNA relative to the germline DNA counterpart, as previously reported[29-31].

### Immunohistochemistry

We obtained 4–6μm thick sections from 140 formalin-fixed and paraffin-embedded (FFPE) samples obtained from surgeries carried out between 1983 and 2025 and sourced from 25 distinct histopathology laboratories; the tissues were fixed and embedded following institution-specific protocols. Sixty-four sections were available as individual slides, 71 were included as cores of four separate tissue microarrays, and 5 samples were available in both forms. After initial optimization, slides were stained with the same anti-RET antibody (Abcam cat#ab134100, EPR2871), a rabbit recombinant monoclonal antibody that recognizes a region at the C-terminus of RET which we have previously shown to detect the two main RET isoforms, known as RET9 and RET51[24]. In brief, antigen retrieval was performed with citrate at pH 6.0 and slides were incubated with the primary antibody for 60 minutes at 35°C with 1:600 or 1:750 dilution for individual slides and TMAs, respectively, and with the secondary antibody for 20 minutes at 37°C. DAB was used for detection, and counterstained with hematoxylin. All steps were performed using the Discovery Ultra IHC stainer (Roche). Slides of a known *RET* and/or *TMEM127-* mutant PCC were used as positive controls in each batch. In addition, 7 slides previously reported[23] were reanalyzed and included. Two observers (RL and FL) blinded to the tumor genotypes evaluated all the slides and recorded a histochemical scoring (H-Score) for both plasma membrane staining (MH-S) and cytoplasm staining (CH-S), calculated based on staining intensity (0 = none, 1 = weak, 2 = moderate and 3 = strong) and the percentage of positively stained cells (P), using the formula[32]: H-score= (0 x P_0_) + (1 x P_1_) + (2 x P_2_) + (3 x P_3_). A total score (TH-S) was obtained by adding MH-S and CH-S. The control tissues were evaluated according to the same intensity scale.

### Statistics

Categorical variables (including tumor location, molecular cluster group and genotype) are reported in absolute frequencies and percentages, while continuous variables are represented as means and standard deviations, median, and ranges. The analysis of group mean differences was conducted using t-tests, multiple paired t-tests, and one-way ANOVA, followed by post-hoc pairwise comparisons utilizing Tukey's Honestly Significant Difference (HSD) test. The receiver operating characteristic (ROC) curve was employed to establish the optimal cutoff and to calculate the sensitivity and specificity of the MH-S and CH-S for identifying *TMEM127* disrupting variants[33]. A 2x2 contingency table was created to outline true positives (TP), false positives (FP), true negatives (TN), and false negatives (FN). The Positive Predictive Value (PPV) was calculated as TP/(TP + FP), while the Negative Predictive Value (NPV) was determined as TN/(TN + FN). For serial testing, we calculated the combined sensitivity and specificity from individual MH-S and CH-S using the following formulas: Serial sensitivity = Sensitivity MH-S * Sensitivity CH-S, and Serial specificity = Specificity MH-S + (1 - Specificity MH-S) * Specificity CH-S. New PPV and NPV values were recalculated based on the results from serial testing. All p-values were two-sided, with a significance level set at p ≤ 0.05. Statistical analyses were performed using RStudio (version 4.5.1, 2025) and GraphPad Prism version 10.0.0 for Windows (GraphPad Software, Boston, Massachusetts, USA).

## Results

Our study cohort included 140 samples from 89 patients with a median age of 35 (range, 1–77 years), 60.6% females (Table 1). Sixty-five patients had PCC, 17 had PGL, 5 had both PCC and PGL, 1 had GIST, and 1 RCC. We performed RET IHC in 140 sections; 19 slides were excluded from subsequent analysis due to technical issues during the staining process, such as tissue detachment from the slides or nuclear staining artifacts. Ten additional slides were excluded because the tissue sections available for staining did not contain sufficient tumor representation. Seven slides of other histologies (5 RCC, one GIST, and one CRC) were not included in the score comparisons of PPGLs. The remaining 104 sections were used for analysis and scoring comparisons. Tissue sections from bilateral adrenal tumors and/or multiple tumors were available from 16 patients. The PPGL samples represented a broad spectrum of genotypes, including both germline and somatic driver events which spanned the three known molecular clusters (Fig. 1, Table 2, below). A total of 38 sections were from cluster 1 (C1) tumors: 17 were in C1A, including *SDHB* (n = 4), *SDHC* (n = 2), *SDHD* (n = 1), *FH* (n = 8); 21 represented C1B: *EPAS1* (n = 12), *VHL* (n = 9). Forty-four sections derived from C2 tumors: *TMEM127* (n = 9), *RET* (n = 16), *RET::GRB2* fusion (n = 1), *HRAS* (n = 2), *MAX* (n = 9), *NF1* (n = 5), and *CSDE1* and *PIK3CA* (n = 1). One sample was from C3, a tumor containing the *UBTF::MAML3* fusion. Additionally, 21 sections were from tumors without a clear driver genetic event, including five samples with *TMEM127* VUS and three with *RET* VUS (Fig. 1, Table 2, below).

### RET expression is higher in pheochromocytomas and kinase signaling tumors

**Figure 2:**
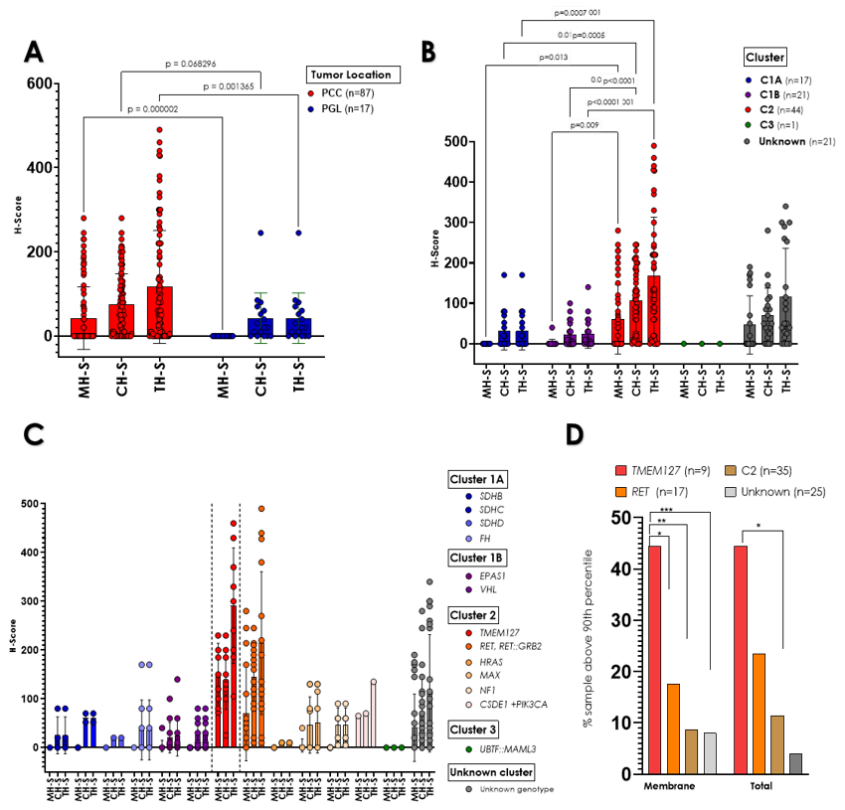
Analysis of RET immunohistochemistry (IHC) H-scores in 104 pheochromocytoma/ paraganglioma sections. A) H-Scores based on tumor site, pheochromocytoma (PCC) or paraganglioma (PGL). Membrane H-Scores (MH-S) in PCC at 42.29±74.18, while in PGL it is 0±0 (p<0.0001), cytoplasm H-score (CH-S) in PCC at 73.98±73.97 versus PGL at 42.27±60.04 (p=0.0682), and total H-Score (TH-S) in PCC at 116.28±133.97 and in PGL at 42.47±60.04 (p=0.001). Multiple t-tests were used to evaluate differences. B) H-Scores categorized by molecular cluster type. For the MH-S, C1A showed 0.0±0.0, C1B 1.90±8.72, C2 60.47±85.82, C3 0±0, and for samples with unknown cluster, 46.61±72.14. For CH-S, C1 31.29±46.80, C1B at 22.85±30.14, C2 at 106.65±77.68, C3 at 0±0, and unknown cluster at 69.23±68.41. For TH-S, C1A 31.29±46.80, C1B 24.76±35.96, C2 167.13±146.08, C3 0±0, and 115.85±120.29 for unknown cluster. Statistical significance was assessed using one-way ANOVA. C) H-scores by tumor genotype. RET-IHC H-Scores of PPGLs stratified by genotype as indicated. Table 3 contains detailed group scores and statistical comparisons. D) Percentage of samples displaying RET IHC H-Scores above the 90th percentile in each of the indicated groups: tumors with TMEM127 pathogenic variants, tumors with RET pathogenic variants, tumors belonging to Cluster 2 (C2, kinase signaling, except for TMEM127-mutant tumors, and samples with unknown genotype (including *RET* VUS and *TMEM127* VUS), distribution of membrane, cytoplasm and total staining. P values were calculated by one-way ANOVA. Comparisons with TMEM127 group are indicated by (*). Other comparisons were not statistically significant.

We first evaluated the distribution of RET scores between PCCs and PGLs of our cohort. RET staining scores were significantly higher in PCCs compared to PGLs (MH-S = 42.29 ± 74.18 vs. 0 ± 0, p < 0.0001, CH-S = 73.98 ± 73.97 vs. 42.47 ± 60.04, p = 0.0682, and TH-S: 116.28 ± 133.97 vs. 42.47 ± 60.04, p = 0.0013, respectively, Table 3, below, Fig. 2A). These findings are in line with previously reported data of RET expression in adrenergic-producing tumors, which comprise a large proportion of PCCs, but are not typically found in PGLs[34-36].

We also evaluated the RET H-Scores by molecular cluster (Fig. 2B). The kinase signaling (C2) tumors exhibited higher total RET scores (167.13 ± 146.08) than those of C1A tumors (31.29 ± 46.80, adj p < 0.0001), C1B tumors (24.76 ± 35.96, adj p < 0.0001) and genetically undetermined PPGLs (115.85 ± 120.29, adj p < 0.001). In addition, CH-S and MH-S were also significantly higher in C2 than in the other groups (Fig. 2B). The C3 group was too small for statistical comparisons. These results are also in agreement with the expected predominance of RET expression in C2/kinase-type PPGLs[6]. Importantly, the format of the sections (individual slide or TMA) did not influence the technical performance or interpretation of the results (Suppl Fig. 1A, B).

#### Tumors carrying pathogenic TMEM127 variants display the highest RET membrane scores

**Figure 3:**
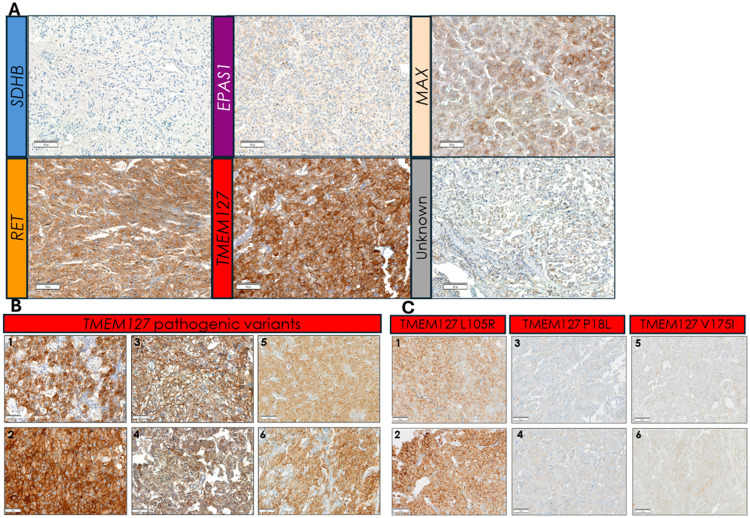
RET IHC sections in tumors with TMEM127-related samples and other genotypes. **A)**Representative examples of RET stained tumors of the indicated genotypes (*SDHB, EPAS1, MAX, RET, TMEM127*, and a tumor of unknown genotype); **B)** RET IHC in tumors with pathogenic *TMEM127* variants: 1, 2, distinct areas of the same tumor; 3 (left), 4 (right) PCCs from the same patient; and 5, 6, distinct regions from the tumor derived from a relative of the patient shown in 3 and 4; **C)** RET IHC in other *TMEM127* variants: 1, 2 two distinct areas of a tumor of unknown genotype carrying a TMEM127 VUS (L105R), showing an area of moderate (1) and one of strong (2) staining; 3, 4, staining of two distinct areas of a PCC with a pathogenic *HRAS* variant and a benign *TMEM127*variant (P18L); 5,6 two separate areas of a PCC with unknown genotype carrying a TMEM127 VUS (V175I). Scale bars are 100μm in A, and 50μm in B and C.

We next focused on tumor-specific genotypes and the performance of tumors with *TMEM127* pathogenic variants. Overall, *TMEM127*-mutant PPGLs showed the highest RET H-Scores of all tested genotypes, displaying generally strong, diffuse distribution with both membrane and cytoplasm localization (Fig. 3A, Fig. 2C, Table 3, below). More specifically, all *TMEM127*-mutant samples had membrane scores above the 75th percentile of the cohort, and 44.4% of them were higher than the 90th percentile (Fig. 2D, Table 2, below). In contrast, 22.8% and 8.5% of the remaining kinase signaling (C2) PPGLs, were above the 75th and 90th percentile, respectively. Total H-S scores were also higher in the TMEM127 group (Fig. 2D), and statistically distinct from C2 PPGLs.

We were particularly interested in the comparison between the TMEM127 group and samples with *RET* variants, given the elevated, albeit variable, reported expression of RET in these tumors[34-36]. We found that *RET-*driven tumors generally displayed cytoplasmic staining (Fig. 2C, Table 3, below), with 64.7% showing scores above the 75th percentile and 35.2% higher than the 90th percentile (Fig. 2D, Suppl Fig. 2A). Only half (9/18) of the RET disrupted samples, including tumors with *RET* pathogenic missense mutations and a *RET* oncogenic fusion, *RET::GRB2*[25], displayed membrane staining (Fig. 2D, Table 2, below). In contrast with the TMEM127 group, only 3 tumors with *RET* pathogenic variants (17.6%) or 4, when also including the tumor with a recombinant fusion (23.5%), had membrane scores above the 90th percentile of the cohort (Table 2, below, Suppl Fig. 2A).

Furthermore, the scores of TMEM127 samples were significantly higher than each of the remaining genotype groups (here, all *SDH*-disrupted samples were combined to allow for statistical comparisons, Suppl Fig. 2B). We also compared the RET IHC scores between *TMEM127* samples and those with unknown driver events. This group also had lower RET membrane and cytoplasm immunoreactivity than TMEM127 samples (MH-S 151.8 ± 62 vs. 40.8 ± 69, respectively, adjusted p = 0.0003, Fig. 2C, Suppl Fig. 2B). Overall, the RET membrane score distribution of TMEM127 samples was significantly different from PPGLs of any other genotype (Table 3, below, Fig. 2D, Suppl Fig. 2A).

The TMEM127 sample group included slides from 7 separate families with distinct variants. While the RET scores varied across samples, we observed relative uniformity across different tumors from the same patient (e.g. bilateral PCCs), as well as from tumors of different individuals from the same family (Fig. 3B, Table 2, below), suggesting sustained stability of RET accumulation, in keeping with the biological role of TMEM127 in RET degradation[23].

#### RET IHC can distinguish tumors carrying functional from those with deleterious TMEM127 variants

In addition to the group of clearly damaging *TMEM127* variants, our cohort also had five other, less well-defined variants in the *TMEM127* gene (Table 4, below). One of these samples was the highest RET-scoring tumor of the group with unknown genotype (total score > 250 in three slides representing bilateral PCCs), from a patient carrying a germline *TMEM127* VUS (c.314T > G, p. L105R, Fig. 3C). Both the clinical features (bilateral, metanephrine-secreting PCCs in a patient older than 60) and detectable loss of wild-type allele in both PCCs (Table 4, below, Suppl Fig. 3A) were consistent with the typical profile of pathogenic *TMEM127* variants, supportive of the L105R variant being deleterious. Therefore, the RET IHC pattern flagged this sample as a potentially dysfunctional *TMEM127* variant.

The remaining four tumors carried *TMEM127* variants that were not classified as pathogenic or likely pathogenic based on the reported literature or ClinVar. These included one PCC classified as VUS (c.523G > T, p.V175F), one classified as benign (c.53C > T, p.P18L), and two other tumors with c.268G > A, p.V90M, classified as likely benign. Two of these tumors had no other identifiable variant, one had a somatic *HRAS*, and the fourth, a *VHL* variant (Table 4, below) and no loss of heterozygosity (Suppl Fig. 3B, 3C). In these four samples, RET staining was low and only detected in the cytoplasm (CH-S ranged from 5–25, MH-S = 0), a pattern markedly distinct from the tumors with damaging *TMEM127* variants (Fig. 3C, Table 2, below).

Overall, these findings suggest that broad and strong RET membrane staining is a consistent feature of tumors with deficient *TMEM127* and this pattern reliably distinguished this group from other PPGL genotypes, including *RET* mutant tumors and tumors carrying nondeleterious *TMEM127* variants.

### RET staining in other tumors

To determine whether RET expression in individuals with *TMEM127* germline pathogenic variants was aberrant in tumors of other histologies, we stained RET in the renal carcinoma of a patient who also carried a pheochromocytoma[37]. In contrast with the strong RET signal of the pheochromocytoma (Suppl Fig. 4A), the renal tumor from the same patient showed no detectable RET staining in tumor cells, typical of the profile of renal cancers generally (Suppl Fig. 4B, 4C), suggesting that RET aberrant expression is likely tissue-specific in *TMEM127*-variant carriers.

#### Evaluation of RET IHC accuracy for identifying PPGLs with deleterious TMEM127 variants

**Figure 4:**
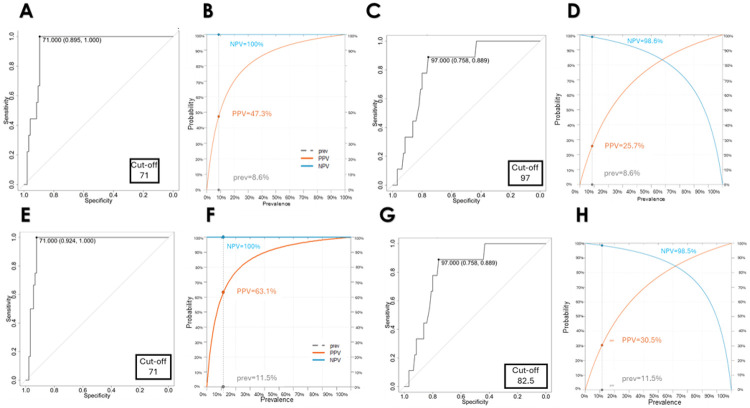
Evaluation of the Test Accuracy. **A)** ROC curve with optimal cutoff of MH-S as indicated, including specificity, and sensitivity to identify *TMEM127* pathogenic tumors; **B)** MH-S curves of PPV and NPV of *TMEM127* cases, with specified prevalence (prev); **C)**ROC curve with optimal cutoff of CH-S, specificity, and sensitivity to identify *TMEM127* cases; **D**) CH-S curves of positive (PPV) (PPV) and negative (NPV) predictive value of TMEM127 cases. **E-H:** same analysis shown in A-D, including one ‘unknown’ tumor with a TMEM127 VUS as a TMEM127 pathogenic variant based on orthogonal validation of the genotype (same tumor shown in Figure 3C-1, 2).

Lastly, we estimated the accuracy of the RET IHC in this cohort using ROC analysis. The prevalence of TMEM127 in this cohort was 8.6%. We established optimal cutoff values for MH-S (71), CH-S (97), and TH-S (178) to effectively identify *TMEM127* cases with a specificity of 89.5% and a sensitivity of 100%. MH-S revealed the highest PPV (47.2%) and NPV (100%) for the *TMEM127* group (Fig. 4A-D). Additionally, inclusion of one additional *TMEM127* VUS sample that had properties of a pathogenic variant in the TMEM127 group (c.314T > G, p. L105R) yielded higher threshold for sensitivity and PPV values, as expected (Fig. 4E-H). Likewise, serial testing of CH-S followed by MH-S improved overall specificity without substantial decrease in sensitivity (Suppl Fig. 5). These analyses support high accuracy of RET IHC as a technically straightforward method for highlighting disruptive *TMEM127* variants.

## Discussion

We recently showed that TMEM127 is an adaptor protein that recruits RET to NEDD4-mediated ubiquitination and degradation by the lysosome[23]. Tumors and cells deficient in TMEM127 function accumulate RET at the cell surface which is reversed by re-expression of intact TMEM127, but not PPGL-associated TMEM127 variants, indicating that RET activation is a key driver of TMEM127-deficient PPGLs[23]. Importantly, TMEM127 deficient tumors were sensitive to RET inhibition[23], suggesting a potential path to treatment for rare cases with aggressive progression[38, 39]. These findings suggest that RET overexpression may serve as a biomarker of *TMEM127* loss of function. Building on these earlier observations, we now report that strong RET membrane expression detected by IHC is a consistent feature of *TMEM127*-mutant tumors, which distinguishes with high specificity and sensitivity this group of samples from other genotypes, including PPGLs driven by *RET* gene disruptions. It remains to be determined why the degree of RET overexpression in PPGLs driven by *TMEM127* variants is greater than that caused by *RET* gain-of-function variants in this assay. However, we note that certain properties of *TMEM127* deficient tumors, such as intact RET sequence and structure, responsiveness to its ligand and co-receptor (GDNF and GFRα), and the considerably extended RET half-life[23] are distinct from mutant *RET*. In addition, whether RET isoform preference[40] and/or distinctive downstream signaling associated with MEN 2A-specific vs MEN 2B-specific *RET* variants[24] contribute to the observed IHC pattern, as well as other phenotypic distinctions between *TMEM127*- and *RET-*driven impact on RET signaling, will require future evaluation.

Importantly, the RET IHC profile was able to effectively discriminate between *TMEM127* variants with nonpathogenic profiles (benign, likely benign and one VUS variant) from a VUS that is likely damaging, based on orthogonal analyses[41], corroborating our findings of known pathogenic variants, and supporting the utility of this assay to infer *TMEM127* function.

In one case, we were also able to find that the RET membrane overexpression in carriers of germline pathogenic TMEM127 variant was limited to PCC but was not detected in another tumor from the same patient (RCC), suggesting that TMEM127’s actions toward RET are likely tissue-specific.

Previous studies have shown that RET immunoreactivity is weak or undetectable in cells of the normal adrenal medulla in both rats and humans, as assessed by IHC[34]. However, PCC derived from patients with hereditary MEN 2A, characterized by *RET* mutations, showed increased RET expression by IHC, and this finding was associated with neuronal differentiation[34, 42, 43]. A few other PPGLs not related to MEN 2 (possibly RET mutation negative) from these earlier cohorts showed RET positive staining; however, the genotype of most of the tumors was unknown. Beyond PPGLs, the application of RET IHC as a biomarker for *RET* mutations and rearrangements that cause gain-of-function alterations in thyroid lesions and lung cancer has not been recommended due to its low sensitivity and high variability in specificity[44]. Therefore, it has been generally considered that the variability of RET staining precluded its adoption as a reliable marker for distinguishing *RET*-mutant from *RET-*intact PPGLs. Here, we confirmed these earlier reports that *RET-* driven tumors often have detectable RET expression by IHC, albeit at variable levels and more frequently localized to the cytoplasm.

The broad genotype representation of our cohort also enabled other comparisons not systematically assessed by IHC previously, revealing increased RET expression in PCCs compared to PGLs, and in tumors belonging to the molecular cluster 2 relative to cluster 1, as previously suspected based on transcription data from human tumors[4, 5] and from PCCs derived from *Nf1* knockout mice[45].

We propose that RET immunohistochemistry, especially when coupled with clinical parameters (PCC localization and metanephrine-predominant biochemical profile) and subcellular distribution of TMEM127[46], serves as a robust tool to identify *TMEM127* disruption in PPGLs of patients with *TMEM127* germline variants.

Limitations of the study: Even though our cohort spanned a broad spectrum of genotypes and approached ‘real world’ status by analyzing samples from a variety of histopathology processing routines in distinct academic or clinical institutions, as well as tissue block age, the number of samples per genotype was still relatively limited, including *TMEM127*-related tumors. However, within this key group we were able to evaluate distinct pathogenic variants, separate tumors from the same individual, or across tumors from different individuals in the same family with highly consistent results. We are also aware that, while pathologists were blinded to the sample genotypes, and that we had two independent pathologists evaluating each of the samples, there is some degree of subjectivity in the intensity scoring. Lastly, we used a single commercial antibody for our investigation and did not compare it with other reagents that may have distinct sensitivity or specificity (for example, to distinct RET isoforms or targeting other epitopes). We therefore anticipate that future studies are warranted to further expand on our observations. Nevertheless, despite these limitations, the robust results suggest that RET IHC can be considered for distinguishing PPGLs with *TMEM127* variants of undefined functional impact. The technical simplicity and broad availability of IHC should make validation studies feasible.

## Supplementary Material

This is a list of supplementary files associated with this preprint. Click to download.

EstradaZunigasupplfigures.pdf

## Figures and Tables

**Table 1:** Summary of the cohort demographics

Age (median, IQR, range)-yr	35, 24–51, 1–77	
Gender (n, %)	Male	29 (32.5)
	Female	54 (60.6)
	U	6 (6.7)
Diagnosis (n, %)	PCC only	63 (70.7)
	PCC + PGL	5 (5.6)
	PCC + RCC	1 (1.1)
	PCC + CRC	1 (1.1)
	PGL only	12 (13.4)
	PGL H&N	4 (4.4)
	PGL + RCC	1 (1.1)
	RCC	1 (1.1)
	GIST	1 (1.1)
Total (n)	89	

IQR:interquartile range, U:unknown, PCC:pheochromocytoma, PGL:paraganglioma, RCC:renal cell carcinoma, CRC:colorectal carcinoma, H&N:head and neck, GIST: gastrointestinal stromal tumor

**Table 2:** RET immunohistochemistry H-Scores of all sections. PCC: Pheochromocytoma; PGL: Paraganglioma; PGL-HN: Paraganglioma Head and Neck; TMA: Tissue Microarray; U: unknown; MH-S: Membrane Histochemical Score; CH-S: Cytoplasm Histochemical Score; TH-S: Total Histochemical Score Histochemical Score; TH-S: Total Histochemical Score

Sample ID	Genotype	Cluster	Tissue	Slide or TMA	MH-S	CH-S	TH-S
UT-1	*TMEM127*	2	PCC	Slide	72	114	186
UT-2	*NF1*	2	PCC	Slide	0	60	60
UT-3	*VHL*	1B	PCC	TMA	0	0	0
UT-4	*VHL*	1B	PCC	TMA	0	10	10
UT-5	*EPAS1*	1B	PGL	TMA	0	60	60
UT-6	U	U	PCC	TMA	70	70	140
UT-7	*TMEM127*	2	PCC	TMA	100	100	200
UT-8	*TMEM127*	2	PCC	TMA	120	120	240
UT-9	*SDHB*	1A	PGL	TMA	0	80	80
UT-10	U	U	PCC	TMA/Slide	0	0	0
UT-11	*VHL*	1B	PCC	TMA	0	0	0
UT-12	*VHL*	1B	PCC	TMA	0	80	80
UT-13	*VHL*	1B	PCC	TMA	0	50	50
UT-14	*VHL*	1B	PCC	TMA	0	30	30
UT-15	U	U	PCC	TMA	0	115	115
UT-16	U	2	PGL-HN	Slide	0	245	245
UT-17	*RET*	2	PCC	Slide	0	80	80
UT-18	*NF1*	2	PCC	Slide	0	20	20
UT-19	*VHL*	1B	PCC	TMA	0	60	60
UT-20	*VHL*	1B	PCC	TMA	0	0	0
UT-21	U	U	PCC	TMA	170	170	340
UT-22	U	U	PCC	TMA	145	145	290
UT-23	U	U	PCC	TMA	0	0	0
UT-24	U	U	PCC	TMA	0	40	40
UT-25	*MAX*	2	PCC	TMA	0	0	0
UT-26	*MAX*	2	PCC	TMA	0	0	0
UT-27	*MAX*	2	PCC	TMA	0	0	0
UT-28	*MAX*	2	PCC	TMA	0	0	0
UT-29	U	U	PCC	TMA	0	50	50
UT-30	U	U	PCC	TMA	50	50	100
UT-31	*SDHB*	1A	PGL	TMA	0	20	20
UT-32	*FH*	1A	PCC	TMA	0	0	0
UT-33	*FH*	1A	PCC	TMA	0	0	0
UT-34	*FH*	1A	PCC	TMA	0	0	0
UT-35	*FH*	1A	PCC	TMA	0	0	0
UT-36	*TMEM127*	2	PCC	TMA/Slide	80	25	105
UT-37	U	1B	PCC	Slide	0	5	5
UT-38	*MAX*	2	PCC	TMA/Slide	40	75	115
UT-39	*MAX*	2	PCC	TMA	0	0	0
UT-40	*SDHD*	1A	PGL	TMA	0	20	20
UT-41	*NF1*	2	PCC	Slide	0	60	60
UT-42	*TMEM127*	2	PCC	TMA	185	185	370
UT-43	*TMEM127*	2	PCC	TMA	150	150	300
UT-44	*SDHC*	1A	PGL	TMA	0	52	52
UT-45	*SDHC*	1A	PGL	TMA	0	70	70
UT-46	*EPAS1*	1B	PCC	Slide	0	0	0
UT-47	*EPAS1*	1B	PCC	Slide	0	30	30
UT-48	*EPAS1*	1B	PCC	Slide	0	30	30
UT-49	*EPAS1*	1B	PCC	TMA	0	0	0
UT-50	*EPAS1*	1B	PCC	TMA	40	100	140
UT-51	*EPAS1*	1B	PGL	TMA	0	0	0
UT-52	*EPAS1*	1B	PCC	TMA	0	0	0
UT-53	*EPAS1*	1B	PCC	TMA	0	0	0
UT-54	U	U	PGL	TMA	0	30	30
UT-55	U	U	PGL-HN	TMA	0	40	40
UT-56	*EPAS1*	1B	PCC	TMA	0	5	5
UT-57	*FH*	1A	PCC	TMA	0	80	80
UT-58	*FH*	1A	PCC	TMA	0	40	40
UT-59	*FH*	1A	PCC	TMA	0	170	170
UT-60	*FH*	1A	PCC	TMA	0	0	0
UT-61	*HRAS*	2	PCC	Slide	0	10	10
UT-62	*UBTF::MAML3 f*usion	3	PGL	Slide	0	0	0
UT-63	U	U	PGL	Slide	0	0	0
UT-64	*RET::GRB2 f*usion	2	PCC	Slide	110	110	220
UT-65	U	U	PGL-HN	Slide	0	85	85
UT-66	*CSDE1/PIK3CA*	2	PCC	Slide	65	70	135
UT-67	U	1A	PCC	Slide	0	0	0
UT-68	*RET*	2	PCC	TMA/Slide	214	214	428
UT-69	*EPAS1*	1B	PGL-HN	Slide	0	10	10
UT-70	*SDHB*	1A	PGL-HN	Slide	0	0	0
UT-71	*SDHB*	1A	PGL	Slide	0	0	0
UT-72	*EPAS1*	1B	PGL	Slide	0	10	10
UT-73	U	1A	PCC	Slide	0	0	0
UT-74	*RET*	2	PCC	Slide	245	245	490
UT-75	U	U	PCC	Slide	0	50	50
UT-76	*TMEM127*	2	PCC	Slide	230	230	460
UT-77	*TMEM127*	2	PCC	Slide	230	200	430
UT-78	*HRAS*	2	PCC	Slide	0	10	10
UT-79	U	U	PCC	Slide	190	110	300
UT-80	U	U	PCC	Slide	175	85	260
UT-81	U	U	PCC	Slide	159	94	253
UT-82	*MAX*	2	PCC	Slide	0	80	80
UT-83	*MAX*	2	PCC	Slide	0	130	130
UT-84	*MAX*	2	PCC	Slide	0	130	130
UT-85	U	U	PCC	Slide	0	15	15
UT-86	*RET*	2	PCC	Slide	50	170	220
UT-87	*RET*	2	PCC	Slide	60	210	270
UT-88	*RET*	2	PCC	Slide	20	200	220
UT-89	U	U	PCC	Slide	20	280	300
UT-90	*RET*	2	PCC	Slide	40	180	220
UT-91	*RET*	2	PCC	Slide	0	110	110
UT-92	*RET*	2	PCC	Slide	0	120	120
UT-93	*NF1*	2	PCC	Slide	0	90	90
UT-94	U	U	PCC	Slide	0	0	0
UT-95	*RET*	2	PCC	Slide	0	90	90
UT-96	*RET*	2	PCC	Slide	0	135	135
UT-97	*RET*	2	PCC	Slide	0	10	10
UT-98	*RET*	2	PCC	Slide	0	20	20
UT-99	*RET*	2	PCC	Slide	0	190	190
UT-100	*RET*	2	PCC	Slide	280	160	440
UT-101	*RET*	2	PCC	Slide	170	210	380
UT-102	*TMEM127*	2	PCC	Slide	200	130	330
UT-103	*NF1*	2	PCC	Slide	0	5	5
UT-104	U	U	PCC	Slide	0	25	25

**Table 3:** RET immunostaining H-Scores distributed by genotype

	SDHB (n = 4)	SDHC (n = 2)	SDHD (n = 1)	FH (n = 8)	EPAS1 (n = 12)	VHL (n = 9)	TMEM127 (n = 9)	RET & RET::GRB2 (n = 17)	HRAS (n = 2)	MAX (n = 9)	NF1 (n = 5)	CSDE1 + PIK3CA (n = 1)	UBTF::MAML3 (n = 1)	UNKNOW (n = 24)
MH-S	0 ± 0	0 ± 0	0 ± 0	0 ± 0	3.33 ± 11.54	0 ± 0	151.88 ± 62.12	69.94 ± 96.87	0 ± 0	4.44 ± 13.33	0 ± 0	65 ± 0	0 ± 0	40.79 ± 69.09
CH-S	25.0 ± 37.85	61.0 ± 12.72	20 ± 0	36.25 ± 61.39	20.41 ± 31.07	26.11 ± 30.39	139.33 ± 61.05	144.35 ± 68.28	10 ± 0	46.11 ± 57.75	47.0 ± 34.2	70 ± 0	0 ± 0	70.79 ± 76.33
TH-S	25.0 ± 37.85	61.0 ± 12.72	20 ± 0	36.25 ± 61.39	23.75 ± 40.96	26.1 ± 30.39	291.22 ± 118.44	214.29 ± 146.40	10 ± 0	50.55 ± 61.66	47.0 ± 34.2	135 ± 0	0 ± 0	111.58 ± 120.23

Pvalues calculated by One-Way ANOVA with Tukey’s multiple comparisons test, related to Fig. 2C.

**Table 4:** Clinical features, genetic, immunofluorescence distribution and RET immunostaining scores of samples with undetermined or nonpathogenic *TMEM127* variants.

Sample ID	Tmem127 variant	Clinical features (age, tumor location, catechol secretion)	Other relevant genetic findings	LOH	Immunofluorescence	ClinVar classification	RET staining (M-HS, C-HS, T-HS)	Combined prediction of dysfunction (damaging/not damaging)
UT-10	c.268G > A, p.V90M	32yo, single nonmetastatic PCC, Epi	No	N	punctate	likely benign	0,0,0	Not damaging
UT-37	c.268G > A, p.V90M	20yo, single nonmetastatic PCC, NMN	*VHL*	N/A	punctate	likely benign	0,5,5	Not damaging
UT-61	c.53C > T, p.P18L	45yo, single nomentastatic PCC, MN	*HRAS*	N/A	N/A	benign	0,10,10	Not damaging
UT-79,80,81	c.314T > G, p.L105R	61yo, bilateral nonmetastatic PCC, MN	No	Y	diffuse	VUS	190,110, 300 (left); 175,85,260 (right)	Damaging
UT-104	c.523G > T, p.V175F	33yo, single nonmetastatic PCC, NMN	No	N	N/A	VUS	0,25,25	Not damaging

PCC:pheochromocytoma; IF:immunofluorescence microscopy pattern of GFP-TMEM127 subcellular distribution (intact TMEM127 has punctate distribution (as reported in^41^); *VHL or *HRAS* pathogenic variants; LOH:loss of heterozygosity; M-HS = membrane scoring; C-HS = cytoplasm scoring; T-HS = total scoring; **two separate blocks from the right pheochromocytoma

Supplementary figures (separate file)

## Data Availability

All data supporting the findings of this study are available within the paper and its Supplementary Information. Additional IHC fiures are available upon request.
